# Feasibility of Predicting the Triglyceride/High-Density Lipoprotein Cholesterol Ratio using Spectral Imaging with a Multi-Material Decomposition Technique on Fast kVp-Switching Dual-Energy CT

**DOI:** 10.2174/0115734056436819260430140356

**Published:** 2026-05-17

**Authors:** Anjie Xie, Chunmei Wang, Shunfa Huang, Jinhui Zhang, Liuhong Zhu, Jianjun Zhou

**Affiliations:** 1 Department of Radiology, Zhongshan Hospital (Xiamen), Fudan University, No.668 Jinhu Road, Huli District, Xiamen 361015, China; 2 Department of Electrocardiography Room, Zhongshan Hospital (Xiamen), Fudan University, No.668 Jinhu Road, Huli District, Xiamen 361015, China; 3 Department of Radiology, Zhongshan Hospital, Fudan University, No. 180 Fenglin Road, Xuhui District, Shanghai 200032, China

**Keywords:** TG/HDL-C, Dual-energy CT, GSI, Nonalcoholic fatty liver disease, Liver fat fraction, Multi-material decomposition

## Abstract

**Introduction/Background:**

Non-alcoholic fatty liver disease (NAFLD) is closely associated with increased cardiovascular risk, and the triglyceride/high-density lipoprotein cholesterol (TG/HDL-C) ratio is a reliable predictor of cardiometabolic disorders. Dual-energy CT with Multi-Material Decomposition (MMD) enables non-invasive quantification of liver Fat Fraction (FF), but its value in predicting TG/HDL-C ratio remains unexplored. To investigate the correlation between the triglyceride/high-density lipoprotein cholesterol (TG/HDL-C) ratio, and spectral parameters, liver Fat Fraction (FF) measured by Multi-Material Decomposition (MMD), and to validate their predictive value.

**Materials and Methods:**

A total of 96 subjects (50 Non-Alcoholic Fatty Liver Disease (NAFLD) patients, 46 healthy individuals) were divided into low-ratio (TG/HDL-C ≤1.5, n=56) and high-ratio (TG/HDL-C >1.5, n=40) groups. Abdominal fast kVp-switching dual-energy CT was performed to measure FF (left lobe (FFL), right anterior lobe (FFRA), right posterior lobe (FFRP)), visceral fat content (FV), virtual monochromatic CT values (HU50-100keV), and liver/spleen CT value ratio (HUL/S). Correlation and ROC analyses were conducted.

**Results:**

TG/HDL-C ratio was positively correlated with FFL, FFRA, FFRP, FV (r=0.7514–0.3787, *p*<0.001) and negatively correlated with HU50-100keV and HUL/S (r=-0.4171–-0.7513, *p*<0.001). The high-ratio group had higher FF and FV, lower HU50-100keV and HUL/S (*p*<0.05). ROC analysis showed the highest AUC was 0.884 (FFRP), followed by FFRA (0.880), FFL (0.872), HUL/S (0.837), and HU100keV (0.835).

**Discussion:**

MMD-derived FF (especially FFRP) exhibited superior predictive performance for TG/HDL-C ratio compared to spectral CT parameters and visceral fat content. This advantage stems from FF’s direct quantification of intrahepatic fat (pathological basis of NAFLD-metabolic interplay), while HU-based parameters are confounded by non-fat tissue components. The study establishes a novel imaging-metabolic association, extending dual-energy CT’s utility from NAFLD diagnosis to cardiovascular risk stratification.

**Conclusion:**

MMD-measured FF and dual-energy CT spectral parameters are correlated with TG/HDL-C ratio, and FF exhibits the best predictive performance for TG/HDL-C in NAFLD patients.

## 1 INTRODUCTION

Non-Alcoholic Fatty Liver Disease (NAFLD) is a metabolic liver disorder characterized by excessive fat accumulation in the hepatic parenchyma. It is closely linked to type 2 diabetes, hyperlipidemia, hypertension, coronary heart disease, and other comorbidities, and may progress to liver cirrhosis or even liver cancer, posing a significant threat to life [1]. Previous studies have confirmed that an elevated triglyceride/high-density lipoprotein cholesterol (TG/HDL-C) ratio serves as an independent predictor of adverse cardiovascular events in both healthy individuals and high-risk populations [2, 3]. Furthermore, the TG/HDL-C ratio demonstrates superior predictive value for adverse cardiovascular events compared to individual lipid parameters or the low-Density Lipoprotein Cholesterol/High-Density Lipoprotein Cholesterol (LDL-C/HDL-C) ratio [4-6].

In recent years, advancements in dual-energy CT spectral technology have introduced tools such as virtual monoenergetic imaging, spectral curves, material separation, and effective atomic number analysis, enabling objective and precise quantitative assessment of fatty liver [7]. Notably, the newly developed multi-material separation technique can quantify hepatic fat content as a volume percentage, a parameter that shows a high correlation with pathological grading [8]. However, existing studies primarily focus on the application of MMD technology in qualitative or semi-quantitative diagnosis of fatty liver, and there is a lack of research exploring its potential in predicting metabolic indicators closely related to cardiovascular risk, such as the TG/HDL-C ratio. To address this research gap, this study is the first to integrate fast kVp-switching dual-energy CT spectral scanning with MMD technology, systematically investigating the correlation between multiple quantitative spectral parameters (including virtual monochromatic CT values, liver-spleen CT value ratio, visceral fat content) and MMD-measured liver Fat Fraction (FF) with the TG/HDL-C ratio in NAFLD patients. This approach addresses the limitation of traditional CT, which supports only qualitative diagnosis of fatty liver, establishes precise associations between imaging parameters and metabolic risk indicators, and provides a rapid, safe, and effective non-invasive method for evaluating cardiovascular disease risk in NAFLD patients.

Despite the clinical significance of TG/HDL-C ratio and advancements in dual-energy CT, critical gaps remain: 1) Existing studies focus on MMD technology for fatty liver qualitative/semi-quantitative diagnosis, but its value in predicting metabolic indicators (*e.g*., TG/HDL-C ratio) linked to cardiovascular risk is unknown; 2) Traditional imaging methods (ultrasound, conventional CT, MRI) lack accuracy in mild steatosis or quantitative metabolic correlation, while invasive liver biopsy has limitations in clinical application. Dual-energy CT + MMD is needed because it enables non-invasive, quantitative measurement of liver fat fraction and multi-dimensional spectral parameters, overcoming the subjectivity and low precision of traditional imaging. This study differs from previous work by systematically exploring the correlation between MMD-derived FF (three liver lobes) and multiple spectral parameters with TG/HDL-C ratio; validating their predictive efficacy *via* ROC analysis; and identifying FF as the optimal predictor, providing a direct imaging-metabolic association for cardiovascular risk assessment in NAFLD patients.

## 2 MATERIALS AND METHODS

### 2.1 Study Population

This retrospective study aimed to investigate 50 patients diagnosed with Non-Alcoholic Fatty Liver Disease (NAFLD) in our institution between 2023 and 2024, who were assigned to the NAFLD group. Among them, 35 males and 15 females were included, with an average age of 52.54 ± 14.17 years.

#### 2.1.1 Inclusion Criteria

The inclusion criteria for the study are as follows:

1) Having undergone a revolution, Computed Tomography (CT) Spectral Imaging (GSI) examination;

2) Meeting the diagnostic criteria specified in the Guidelines for the Diagnosis and Treatment of Non-Alcoholic Fatty Liver Disease [9], with diagnosis confirmed based on the comprehensive assessment by clinicians.

#### 2.1.2 Exclusion Criteria

The exclusion criteria are as follows:

1) A history of excessive alcohol consumption (ethanol intake ≥ 140 g/week for males and ≥ 70 g/week for females);

2) Patients with viral hepatitis, drug-induced liver disease, malignant space-occupying lesions of the liver, autoimmune liver disease, or other conditions that may cause NAFLD or affect the results of abdominal CT examination.

Additionally, 46 healthy individuals who underwent physical examination during the same period were selected as the control group, including 19 males and 27 females, with an average age of 47.74 ± 12.67 years. All individuals in the control group were excluded from NAFLD by GSI examination, and other liver diseases were ruled out based on medical history and relevant examinations.

### 2.2 Methods

#### 2.2.1 GSI Examination

##### 2.2.1.1 Examination Method

Abdominal plain scanning was performed using a Revolution CT scanner (Revolution CT, GE Healthcare, USA). The scanning parameters were as follows: GSI mode; pitch 0.508; rotation speed 0.8 s/r; detector width 80 mm; instantaneous (0.5 ms) switching between 140–80 kVp; tube current 320–400 mA; slice thickness 5 mm; slice interval 5 mm; reconstruction method: 50% adaptive statistical iterative reconstruction (ASIR-V); and reconstructed Date images with a slice thickness of 1.25 mm.

##### 2.2.1.2 Image Processing and Data Measurement

After the scan was completed, the Date images were uploaded to the GE AW4.6 workstation. Two radiologists (with 5 and 3 years of diagnostic experience, respectively) performed image measurements in a double-blind manner. For Fat Fraction (FF) measurement, the “Liver fat” analysis option was selected. The reference level was defined as the axial slice at the point where the right portal vein branch enters the liver (identified by two radiologists independently; in case of disagreement, a third radiologist with 10 years of experience was consulted for arbitration). Three Regions of Interest (ROIs) were selected in the left lobe, right anterior lobe, and right posterior lobe of the liver: 1) Left lobe ROI: Located 2.0 ± 0.2 cm away from the liver edge, avoiding the left hepatic vein and ligamentum teres hepatis; 2) Right anterior lobe ROI: Adjacent to the gallbladder fossa, 1.5 ± 0.2 cm away from the nearest portal vein branch, avoiding the middle hepatic vein; 3) Right posterior lobe ROI: Midway between the right hepatic vein and the liver capsule, 1.0–1.5 cm away from the liver surface. The diameter of each ROI was standardized to 15 ± 1 mm, with strict avoidance of hilar blood vessels, bile ducts, intra-abdominal adipose tissue, and artifacts. Inter-observer and intra-observer agreement were evaluated using the Intraclass Correlation Coefficient (ICC). The ICC values for FF measurements were 0.93 (95% CI: 0.89–0.96) for inter-observer agreement and 0.95 (95% CI: 0.92–0.97) for intra-observer agreement, confirming high reproducibility. The fat fraction percentage of each ROI was obtained, and the average of three measurements for each lobe was taken as the final result of liver fat content, namely FFL (Fat Fraction of Left Lobe), FFRA (Fat Fraction of Right Anterior Lobe), and FFRP (Fat Fraction of Right Posterior Lobe).

Software algorithm: The “Liver fat” module (version 2.1) on the GE AW4.6 workstation adopts a three-Material Decomposition (MMD) algorithm that segregates liver tissue into three components (fat, water, and protein) based on their distinct X-ray attenuation characteristics at dual energies (80 kVp and 140 kVp). Fat fraction is calculated as: FF (%) = (Volume of fat component / Total volume of fat + water + protein components) × 100%.

Thresholding: For visceral fat content (FV) and visceral fat percentage (FCV) measurement, the “Reformat” function was used with a Hounsfield Unit (HU) threshold of -190 to -30. This range specifically targets adipose tissue while excluding non-adipose structures (*e.g*., muscles, organs, blood vessels).

Anatomical boundaries: Visceral fat was defined as adipose tissue enclosed within the parietal peritoneum and abdominal muscle fascia (anatomical boundary between visceral and subcutaneous fat). Specifically, visceral fat includes adipose tissue surrounding the liver, spleen, stomach, intestines, and other intra-abdominal organs; subcutaneous fat located outside the abdominal muscle fascia was excluded from measurement.

By selecting the “Monoenergetic” option and choosing the maximum slice of the spleen, the monoenergetic CT values of the ROI at 40 keV, 50 keV, 70 keV, and 100 keV, as well as the liver/spleen CT value ratio at 70 keV (HUL/S), were obtained. The Reformat function was used to measure visceral fat content (FV) and visceral fat content percentage (FCV).

#### 2.2.2 Collection of Clinical Data

Data such as gender, age, and medical history of the patients were recorded. Height and weight were measured to calculate Body Mass Index (BMI), and lipid profiles were routinely tested.

#### 2.2.3 Grouping

Participants were divided into two groups based on TG/HDL-C ratio: the low tertile group (≤1.5; 56 cases) and the high tertile group (>1.5; 40 cases).

### 2.3 Statistical Methods

Statistical analyses were performed using SPSS 19.0 software. Normality of continuous variables (FFL, FFRA, FFRP, FV, FCV, HU50keV, HU70keV, HU90keV, HU100keV, HUL/S, and TG/HDL-C ratio) was tested using the Shapiro-Wilk test. The test results showed that all variables had a Shapiro-Wilk statistic > 0.90 (*p* > 0.05), indicating a normal distribution. Thus, a two-sample t-test was used to compare differences between the low-ratio and high-ratio groups for normally distributed continuous variables. Pearson's correlation analysis was used to assess the linear relationship between each CT parameter and the TG/HDL-C ratio. Receiver Operating Characteristic (ROC) curve analysis was used to evaluate the efficacy of each indicator in predicting the TG/HDL-C ratio. Pairwise comparison of AUC values was performed using the DeLong test. A *p*-value < 0.05 was considered statistically significant.

## 3 RESULTS

### 3.1 Comparison of FFL, FFRA, FFRP, FV, and FCV between the Two Groups

The levels of FFL, FFRA, FFRP, and FV in the low tertile group were significantly lower than those in the high tertile group, with statistically significant differences (*P* < 0.05), as shown in Table **1**.

### 3.2 Comparison of HU50keV, HU70keV, HU90keV, HU100keV, and HUL/S between the Two Groups

The levels of HU50keV, HU70keV, HU90keV, HU100keV, and HUL/S in the low tertile group were significantly higher than those in the high tertile group, with statistically significant differences (*P* < 0.05), as shown in Table **2**.

### 3.3 Correlation Analysis

Correlation analysis showed that the TG/HDL-C ratio was positively correlated with FFL, FFRA, FFRP, FV, and FCV, with correlation coefficients of 0.7514 (95% Confidence Interval (CI): 0.6452 to 0.8292, *p* < 0.0001), 0.7502 (95% CI: 0.6435 to 0.8283, *p* < 0.0001), 0.7469 (95% CI: 0.6391 to 0.8259, *p* < 0.0001), 0.3787 (95% CI: 0.1918 to 0.5391, *p* = 0.0002), and 0.4105 (95% CI: 0.2231 to 0.5686, *p* < 0.0001), respectively, as shown in Fig. (**1**).

In contrast, the TG/HDL ratio was negatively correlated with HU50keV, HU70keV, HU90keV, HU100keV, and HUL/S, with correlation coefficients of -0.4171 (95% CI: -0.5740 to -0.2307, *p* < 0.0001), -0.6129 (95% CI: -0.7272 to -0.4655, *p* < 0.0001), -0.6878 (95% CI: -0.7830 to -0.5612, *p* < 0.0001), -0.7006 (95% CI: -0.7923 to -0.5778, *p* < 0.0001), and -0.7513 (95% CI: -0.8291 to -0.6450, *p* < 0.0001), respectively, as shown in Fig. (**2**).

### 3.4 ROC Analysis

ROC analysis showed that the Area Under the Curve (AUC) values of FFL, FFRA, FFRP, and FV for predicting the TG/HDL-C ratio were 0.872, 0.880, 0.884, and 0.757, respectively (all *P* < 0.05). For HU50keV, HU70keV, HU90keV, HU100keV, and HUL/S, the AUC values for predicting the TG/HDL-C ratio were 0.723, 0.803, 0.832, 0.835, and 0.837, respectively (all *P* < 0.05). Pairwise comparison of AUC values was performed using the DeLong test to verify the statistical superiority of FF. The results showed that: 1) FFRP (AUC=0.884) had a significantly higher AUC than non-FF parameters including HUL/S (0.837, p=0.032), HU100keV (0.835, p=0.028), FV (0.757, *p*<0.001), and HU50keV (0.723, *p*<0.001); 2) No significant difference was found between FFRP and other FF parameters (FFRA: 0.880, p=0.761; FFL: 0.872, p=0.583); 3) FFRA (AUC=0.880) was also significantly superior to FV (*p*<0.001) and HU50keV (*p*<0.001), with no statistical difference from HUL/S (p=0.064) or HU100keV (p=0.059).

These findings confirm that MMD-measured FF (especially FFRP) is statistically superior to non-FF spectral parameters and visceral fat content in predicting TG/HDL-C ratio, while the three FF subparameters (FFL, FFRA, FFRP) have comparable predictive efficacy. Details are shown in Table **3**.

### 3.5 CT Features of Different TG/HDL-C Ratios

**Fig. (3):**
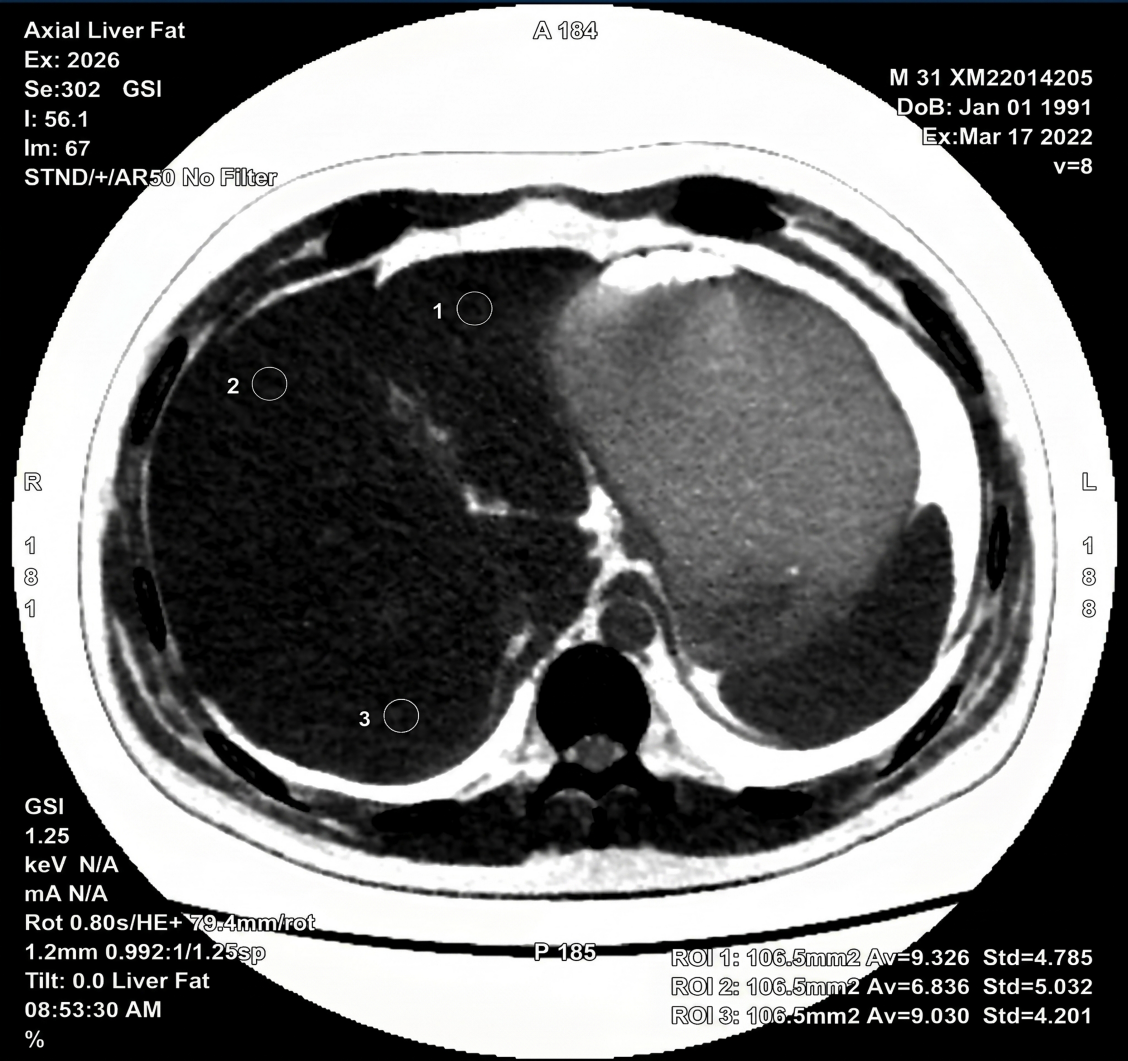
High TG/HDL-C ratio group (liver fat fraction image).

Figure **3** High tertile group: A 31-year-old male. At this level, the measurements were 9.326 (left hepatic lobe), 6.836 (right anterior hepatic lobe), and 9.030 (right posterior hepatic lobe).

**Fig. (4):**
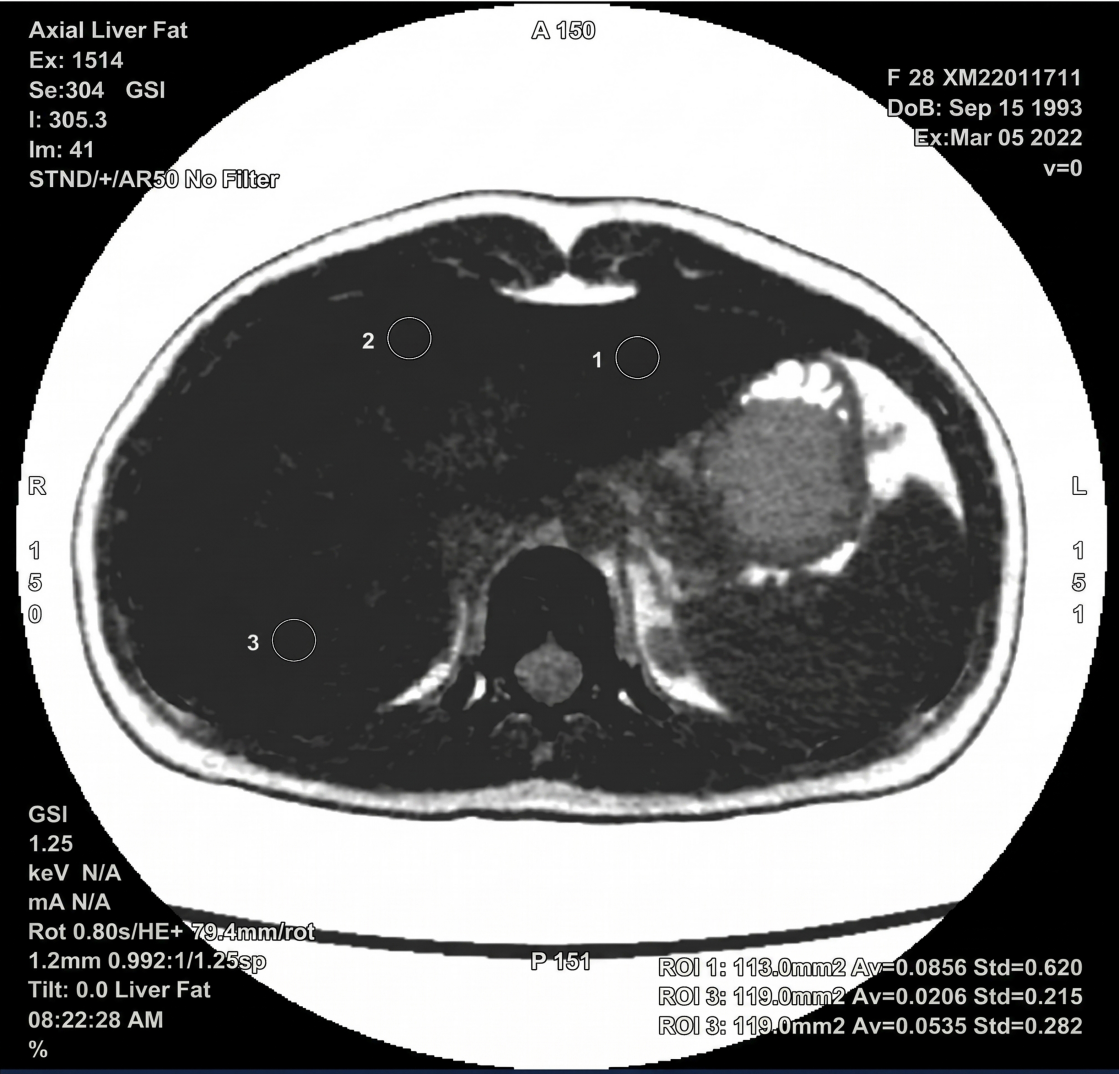
Low TG/HDL-C ratio group (liver fat fraction image).

Figure **4** Low tertile group: A 28-year-old female. At this level, the measurements were 0.0856 (left hepatic lobe), 0.0206 (right anterior hepatic lobe), and 0.0535 (right posterior hepatic lobe).

**Fig. (5):**
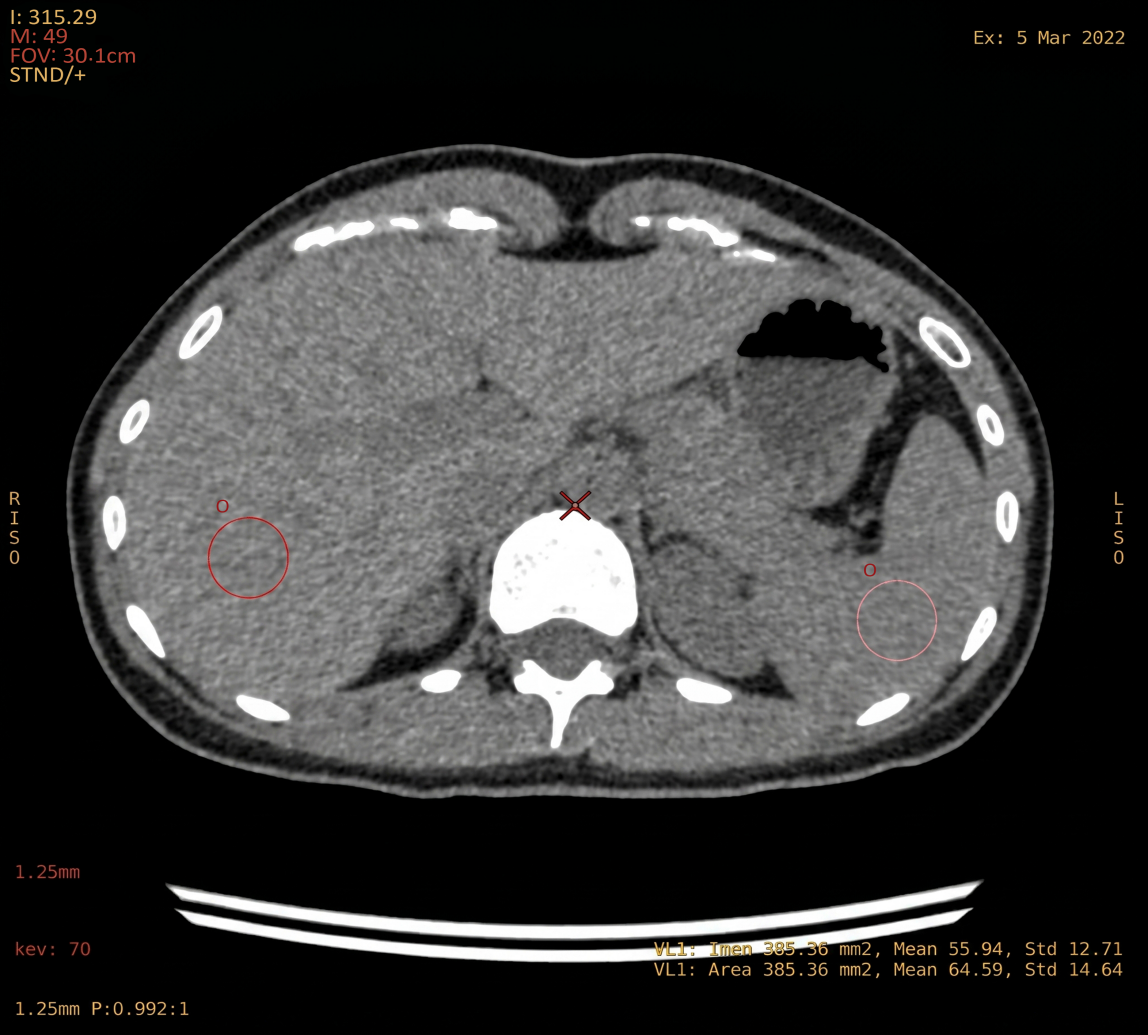
High TG/HDL-C ratio group (70 keV monochromatic image).

Figure **5** High tertile group: At 70 keV, the CT value of the liver at this level was measured as 55.94 Hu, and the CT value of the spleen was 64.59 Hu.

**Fig. (6):**
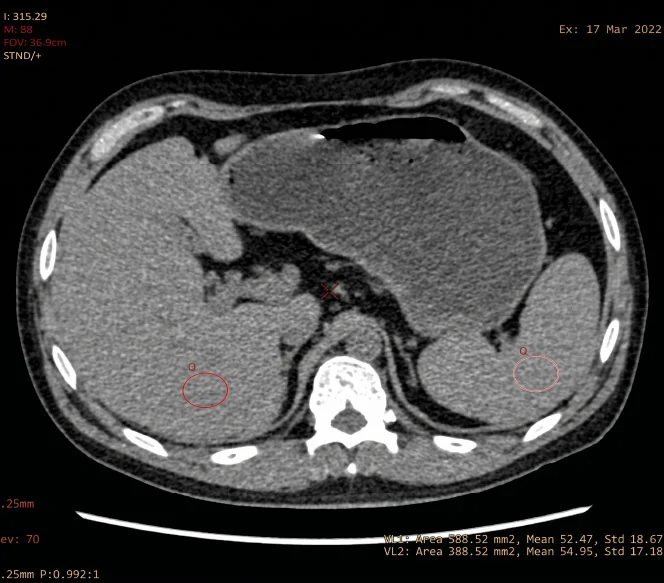
Low TG/HDL-C ratio group (70 keV monochromatic image).

Figure **6** Low tertile group: At 70 keV, the CT value of the liver at this level was measured as 52.47 Hu, and the CT value of the spleen was 54.95 Hu.

## 4 DISCUSSION

Patients with Non-Alcoholic Fatty Liver Disease (NAFLD) are at an elevated risk of cardiovascular diseases; however, there remains a paucity of safe and effective predictive modalities. This study employed dual-energy CT spectral imaging combined with Multi-Material Decomposition (MMD) technology to investigate the association between CT quantitative parameters and the Triglyceride/High-Density Lipoprotein Cholesterol (TG/HDL-C) ratio. Research conducted by Sam *et al* [10]. demonstrated that healthy adults exhibited a significant increase in cardiovascular disease risk following short-term weight gain induced by a high-energy diet. With equivalent weight gain, a higher proportional increase in CT-quantified visceral fat area was correlated with more severe arteriosclerotic changes. This phenomenon may be attributed to the fact that intra-abdominal fat, distributed predominantly within the abdominal cavity, participates in the organism's endocrine and metabolic functions. Intra-abdominal adipose tissue is composed of large-sized, insulin-resistant adipocytes, whose metabolic by-products directly enter the portal venous system, accompanied by heightened catabolic activity of adipocytes.

In the present study, visceral fat content (FV) in the high tertile group was significantly higher than in the low tertile group (*P* < 0.05). Pearson correlation analysis revealed a positive correlation between FV and the TG/HDL-C ratio (*P* < 0.05), thereby confirming a close association between intra-abdominal fat area and the TG/HDL-C ratio. Previous studies have shown that, compared with conventional CT images, virtual monoenergetic images generated by Revolution CT mitigate beam-hardening artifacts and enhance lesion visualization quality [11]. Animal experimental studies have confirmed that monoenergetic CT values at 70 keV can be utilized to differentiate between various pathological grades of fatty liver [12]. Nevertheless, research regarding the diagnostic efficacy of CT values at different keV levels for adult fatty liver remains limited.

In this study, HU50keV, HU70keV, HU90keV, HU100keV, and hepatic-to-splenic CT value ratio (HUL/S) were recorded in patients with fatty liver. The analytical results indicated that HU50keV, HU70keV, HU90keV, HU100keV, and HUL/S in the low tertile group were higher than those in the high tertile group; the TG/HDL ratio exhibited a negative correlation with HU50keV, HU70keV, HU90keV, HU100keV, and HUL/S. Among these parameters, HU100keV yielded the highest Area Under the Curve (AUC) value (0.835) for predicting the TG/HDL-C ratio, which was comparable to the AUC value of HUL/S (0.837).

The “Liver fat” analysis software was utilized in this study to quantify liver fat content. The results showed that the fat fractions of the left lobe (FFL), right anterior lobe (FFRA), and right posterior lobe (FFRP) were lower in the low tertile group than in the high tertile group. Correlation analysis further revealed a positive correlation between the TG/HDL-C ratio and FFL, FFRA, and FFRP. By analyzing the correlations among FFL, FFRA, FFRP, FV, visceral fat content percentage (FCV), and the TG/HDL-C ratio, it was confirmed that the “Liver fat” analysis function of Revolution CT accurately reflects the severity of fatty liver. Additionally, most previous studies used the mean fat content of three regions of interest (ROIs) in the left lobe, right anterior lobe, and right posterior lobe as the measurement of liver fat content. In the current study, ROC analysis demonstrated that FFRP achieved the highest AUC value (0.884) for predicting the TG/HDL-C ratio. Whether the sole utilization of FFRP as a measurement parameter confers greater advantages in the diagnosis of fatty liver necessitates further verification and exploration in subsequent studies.

At present, liver biopsy remains the “gold standard” for diagnosing NAFLD. However, as an invasive examination, it may cause complications such as bleeding and infection. Moreover, it is not widely accepted in clinical practice due to sampling errors at the puncture site and inter-observer variability [13]. Ultrasound (US), conventional Computed Tomography (CT), and Magnetic Resonance Imaging (MRI) are the most commonly used imaging methods for diagnosing NAFLD. Ultrasound is simple to operate, cost-effective, and radiation-free, but it lacks quantitative standards and is greatly affected by the operator's subjectivity [14]. MRI can use a variety of imaging techniques such as Magnetic Resonance Spectroscopy (MRS), chemical shift imaging, and fat saturation imaging. However, most of these techniques are only suitable for qualitative or semi-quantitative diagnosis and are easily affected by iron deposition [15]. Conventional CT usually uses liver CT values to measure fat content. Studies have confirmed that the liver/spleen CT value ratio in NAFLD patients has a high consistency with the results of liver pathological diagnosis. However, it lacks accuracy in diagnosing NAFLD with steatosis < 30% [16]. In addition, liver CT values are also easily affected by hepatic iron and the intake of iodine-containing drugs.

Furthermore, due to the large variation in spleen CT values, the accuracy of quantitative analysis of NAFLD is inevitably affected [17]. To sum up, imaging results lack accuracy in populations with mild hepatic steatosis. Therefore, this study included healthy populations and populations with non-alcoholic fatty liver disease, using a broader population to verify the universality of the results.

When the Triglycerides (TG) synthesized in hepatocytes exceed the uptake and utilization capacity of extrahepatic tissues, the serum TG level increases, leading to intrahepatic TG deposition. TG is the main factor contributing to lipid accumulation in fatty liver. Therefore, the serum TG level can be a sensitive indicator of the severity of fatty liver lesions [18]. High-Density Lipoprotein Cholesterol (HDL-C) is composed of apolipoproteins, phospholipids, cholesterol, and a small amount of fatty acids, and it can reversely transport Total Cholesterol (TC) to the liver. Under the action of cholesteryl ester transfer protein, lipid exchange occurs between TG in lipoproteins and cholesterol in low-density lipoprotein (LDL-C) and HDL-C. The higher the TG level, the more active the exchange, leading to increased LDL-C concentration, decreased HDL-C levels, elevated TG-rich lipoprotein remnants, and increased cholesterol content [19]. Previous studies have confirmed that HDL is an important protective factor against fatty liver, and a decrease in HDL levels can significantly increase the risk of developing fatty liver [20]. Thus, the TG/HDL-C ratio can more comprehensively reflect the intrinsic composition and content of triglyceride lipoproteins compared to the single TG index, and changes in lipid indicators are closely associated with Non-Alcoholic Fatty Liver Disease (NAFLD).

### 4.1 Comparison with Previous Studies

To contextualize our findings, we systematically contrast them with 5 recent relevant studies focusing on dual-energy CT, MMD technology, or TG/HDL-C ratio-related metabolic risk assessment:

(1) Wang *et al.* (2024) [7] reported that multi-parameter spectral CT imaging could quantitatively measure fatty liver severity but did not explore the correlation between these imaging parameters and metabolic indicators (*e.g*., TG/HDL-C ratio) linked to cardiovascular risk. In contrast, the current study establishes a direct, quantitative association between MMD-derived FF, spectral parameters, and the TG/HDL-C ratio, extending the clinical value of spectral CT beyond fatty liver diagnosis to metabolic risk prediction.

(2) Dandan *et al.* (2021) [8] focused on the impact of hepatic iron deposition on the accuracy of MMD-measured FF, but did not involve clinical metabolic parameters or cardiovascular risk indicators. This study excludes patients with iron deposition-related liver diseases *via* strict inclusion criteria, and further validates FF’s predictive efficacy for TG/HDL-C ratio, expanding the clinical application scenario of MMD technology in metabolic risk assessment.

(3) Cao et *al.* (2019) [12] validated the value of multi-parameter dual-energy CT in diagnosing NAFLD in rat models, but lacked human cohort data on metabolic correlation. The study is based on 96 human subjects (including NAFLD patients and healthy controls), demonstrating the translational value of FF and spectral parameters in predicting TG/HDL-C ratio in clinical populations.

(4) Gun *et al.* (2020) [21] validated that quantitative CT could accurately measure liver fat content by comparing with MRI Proton Density Fat Fraction (PDFF), but did not assess the predictive value for TG/HDL-C ratio. The ROC analysis shows that MMD-measured FF (especially FFRP; AUC=0.884) outperforms traditional CT parameters (*e.g*., HUL/S; AUC=0.837) in predicting the TG/HDL-C ratio, highlighting the superiority of integrating MMD technology into metabolic risk assessment.

(5) Choi *et al.* (2018) [22] confirmed a correlation between visceral fat volume (measured by CT) and hepatic steatosis (measured by MRI), but did not involve TG/HDL-C ratio or MMD-derived FF. This study identifies FF as a stronger predictor of TG/HDL-C ratio than visceral fat content (FV, AUC=0.757 vs. FFRP=0.884), providing a more precise and specific imaging marker for metabolic risk stratification in NAFLD patients.

### 4.2 Why FF Performs Better than HU-based Predictors

The superior predictive performance of FF over HU-based predictors (HU50-100keV, HUL/S) can be attributed to three key factors: 1) Mechanistic specificity: FF directly quantifies intrahepatic fat volume percentage *via* MMD technology, which is the pathological basis of NAFLD and closely linked to TG synthesis and metabolism. In contrast, HU-based parameters indirectly reflect tissue attenuation, which can be affected by factors such as hepatic iron, iodine intake, and variability in spleen function. 2) Quantitative accuracy: The MMD algorithm decomposes liver tissue into fat, water, and protein, eliminating the interference of non-fat components on measurement results. HU values, however, are influenced by beam hardening artifacts and tissue composition heterogeneity, leading to lower precision in mild-to-moderate steatosis. 3) Regional specificity: FF measurements from three liver lobes (especially FFRP) capture the spatial distribution of hepatic fat, which is more closely associated with systemic lipid metabolism (*e.g*., TG/HDL-C ratio) than global attenuation-based HU parameters. These advantages make FF a more direct and reliable predictor of the TG/HDL-C ratio.

### 4.3 Study Limitations

#### 4.3.1 The Limitations of this Study are as Follows

(1) Limitation of retrospective design: A retrospective analysis was adopted, and data collection relied on previous diagnosis and treatment records. Although biases were reduced through strict inclusion and exclusion criteria, selection bias may still exist (*e.g*., only patients with complete data were included). Additionally, data on potential confounding factors such as the lifestyle (*e.g*., diet, exercise) of the subjects could not be obtained, which may affect the accuracy of the results.

(2) Limitation of sample size and representativeness: The total sample size was 96 cases. Although it met the minimum sample size requirement calculated earlier, the sample remained small, and all samples came from a single medical institution. The age and gender composition of the subjects may limit generalizability, so caution should be exercised when extrapolating the results to other populations (*e.g*., populations of different ethnicities or people in primary hospitals).

(3) Limitations of measurement and technology: Although CT scanning parameters were set uniformly, differences in scanning phases (*e.g*., respiratory status) among different patients may exist, which could affect the stability of ROI measurement; currently, most studies on the diagnosis of NAFLD using Revolution CT are based on animal experimental models [21, 22], which have certain limitations.

(4) Limitations of retrospective design and lack of histopathology validation: This study is retrospective, which may introduce selection bias (*e.g*., only patients with complete data were included). Additionally, due to the non-invasive nature of the study, we did not perform a liver biopsy for histopathology validation. Although MMD-derived FF has been previously validated against pathological results [8,22], direct histopathological correlation with TG/HDL-C ratio is lacking. Future prospective studies with histopathology confirmation are needed to further validate our findings.

## CONCLUSION

In conclusion, the quantitative parameters (FV, HU50keV, HU70keV, HU90keV, HU100keV, HUL/S) provided by dual-energy CT spectral scanning with fast kVp switching, as well as FFL, FFRA, and FFRP measured by MMD technology, are all correlated with the TG/HDL-C ratio. These parameters can be used to predict TG/HDL-CLFC expression in NAFLD. Among them, FF (Fat Fraction) shows the best performance. This can provide a new objective and quantitative basis for the early screening of NAFLD and dynamic monitoring of treatment efficacy, and promote the comprehensive management of cardiovascular disease risk factors in patients. Obtaining more information through spectral CT can be an important means of screening for cardiovascular diseases, such as atherosclerotic plaques, in NAFLD patients.

## Figures and Tables

**Fig. (1):**
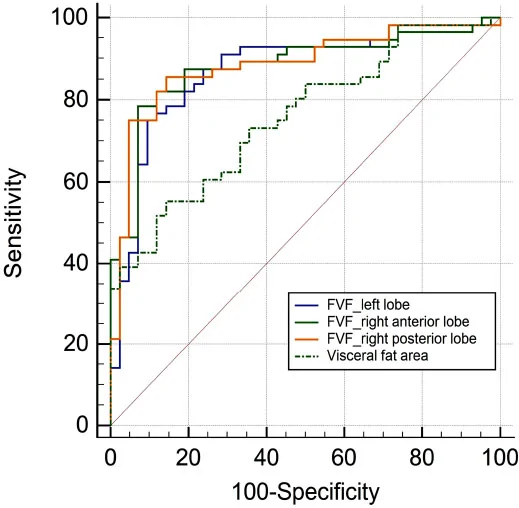
Positive correlations between TG/HDL-C ratio and MMD-Derived liver fat Fractions/Visceral fat content.

**Fig. (2):**
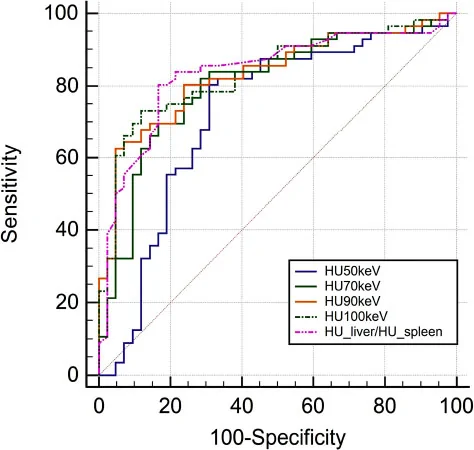
Negative correlations between TG/HDL-C ratio and virtual monochromatic CT Values/Liver-Spleen CT ratio (HUL/S).

**Table 1:** Comparison of FFL, FFRA, FFRP, FV, and FCV between the two groups.

**Parameters**	**TG/HDL-C**		
	**Low (<=1.5)**	**High (>1.5)**	**P**
FF_L_	1.3516±3.16	6.7548±5.77	*p*<0.0001
FF_RA_	1.3822±3.56	6.2228±5.62	*p*<0.0001
FF_RP_	2.1497±4.07	8.6130±5.20	*p*<0.0001
FV	12610.84±6224.39	18541.85±5790.94	*p*<0.0001
FCV	60.66±11.46	64.45±11.41	P=0.097

**Table 2:** Comparison of HU50keV, HU70keV, HU90keV, HU100keV, and HUL/S between the two groups parameters.

**Parameters**	**TG/HDL-C**		
	**Low (<=1.5)**	**High (>1.5)**	**P**
HU_50keV_	71.02±10.23	63.55±13.31	*p*<0.0001
HU_70keV_	62.38±7.99	53.52±8.65	*p*<0.0001
HU_90keV_	58.91±7.46	49.51±7.68	*p*<0.0001
HU_100keV_	57.91±7.39	48.40±7.57	*p*<0.0001
HU_L/S_	1.17±0.17	0.97±0.16	*p*<0.0001

**Table 3:** ROC analysis.

	**AUC**	**Threshold**	**Sensitivity**	**Specificity**	**Yoden Index**
FF_L_	0.872	0.898	0.905	0.750	0.655
FF_RA_	0.880	1.065	0.929	0.786	0.715
FF_RP_	0.884	5.109	0.857	0.857	0.714
FV	0.757	13013	0.857	0.544	0.411
HU_50keV_	0.723	68.625	0.804	0.69	0.494
HU_70keV_	0.803	57.71	0.821	0.714	0.535
HU_90keV_	0.832	58.785	0.625	0.952	0.577
HU_100keV_	0.835	55.89	0.732	0.881	0.613
HU_L/S_	0.837	1.105	0.804	0.833	0.637

## Data Availability

The data and supportive information are available within the article.

## LIST OF ABBREVIATIONS

TG/HDL-C: Triglyceride/High-Density Lipoprotein Cholesterol
FF: Fat Fraction
MMD: Multi-Material Decomposition
NAFLD: Non-alcoholic fatty liver disease
FV: Fat content
TG: Triglycerides
HDL-C: High-Density Lipoprotein Cholesterol
TC: Total Cholesterol
LDL-C: Low-density lipoprotein

## STANDARDS OF REPORTING

STROBE guidelines were followed.
